# Computational modelling approach for the optimization of apple juice clarification using immobilized pectinase and xylanase enzymes

**DOI:** 10.1016/j.crfs.2020.09.003

**Published:** 2020-10-14

**Authors:** Shady S. Hassan, Gwilym A. Williams, Amit K. Jaiswal

**Affiliations:** a School of Food Science and Environmental Health, College of Sciences and Health, Technological University Dublin - City Campus, Grangegorman, Dublin 7, Ireland; b Environmental Sustainability and Health Institute, Technological University Dublin - City Campus, Grangegorman, Dublin 7, Ireland; c School of Biological and Health Sciences, College of Sciences and Health, Technological University Dublin, Kevin Street, Dublin 8, Ireland

**Keywords:** Central composite rotatable design (CCRD), Artificial neural network (ANN), *Mucor hiemalis*, Pectinase, Xylanase, Alginate beads, Apple juice clarification

## Abstract

Apple juice is typically marketed as a clear juice, and hence enzymatic treatments are common practices in juice industry. However, enzymatic treatments have been shown to face some challenges when process efficiency, and cost effectiveness are concerned. Therefore, it is necessary to optimize the enzymatic treatment process to maximize efficiency, and reuse enzymes to minimize the overall cost via immobilization. In this context, the present work features the immobilization of pectinase and xylanase from *M. hiemalis* on genipin-activated alginate beads, with subsequent evaluation of its efficacy in apple juice clarification. A central composite rotatable design (CCRD), coupled with artificial neural network (ANN) for modeling and optimization was used to design the experiments. Deploying a coupling time up to 120 min, and an agitation rate of 213 rpm (pectinase) - 250 rpm (xylanase), a maximum fractional enzyme activity recovered was observed to be about 81–83% for both enzymes. Optimum enzyme loading and genipin concentration were found to be 50 U/ml and 12% (w/v), respectively. The immobilized enzyme preparations were suitable for up to 5 repeated process cycles, losing about 45% (pectinase) - 49% (xylanase) of their initial activity during this time. The maximum clarity of apple juice (%T_660_, 84%) was achieved at 100 min when pectinase (50 U/ml of juice) and xylanase (20 U/ml of juice) were used in combination at 57 °C. The immobilized enzymes are of industrial relevance in terms of biocompatibility, recoverability, and operational-storage stability.

## Highlights

• Computational modeling adopted for enzymes immobilization and juice clarification.
• Maximum recovery of enzyme activity of pectinase was ~81% and ~83% for xylanase.
• Immobilization improved storage and operational stability of pectinase and xylanase.
• Immobilized enzymes retained ~50% of its initial activity even after five cycles.
• Immobilized pectinase and xylanase together showed apple juice clarification of ~84%.

## Graphical abstract

**Figure:**
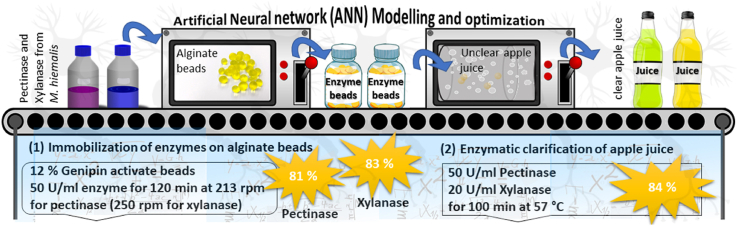

## 1 Introduction

Unlike animal counterparts, plant cells are surrounded by an extracellular matrix known as the cell wall which comprises polysaccharide and protein polymers. Protein accounts for only 5–10% of this structure, whereas polysaccharides constitute 90–95% of the cell wall (Jacq et al., 2017). Such polysaccharides are predominantly pectin, hemicellulose, and cellulose (Broxterman and Schols, 2018). In a typical cell of hardwood, such as apple, the wall possesses high pectin levels, and the predominant hemicellulose fraction is xylan (Donev et al., 2018). However, structural non-cellulosic polysaccharides, such as pectin and xylan, are indigestible by human digestive enzymes in the upper gastrointestinal tract (Tappia et al., 2020). Additionally, the presence of colloidal particles of pectin and xylan results in an undesirable cloudiness in apple juice (Kuddus, 2018). Hence, pectinase and xylanase enzymes have been commonly applied to clarification in the apple juice industry (Garg et al., 2016; Nagar et al., 2012; Sharma and PatelSugandha, 2017).

A key concern with the use of enzymes in cost-sensitive food processing operations is the relative expense of such biocatalysts. In this context, immobilization technology affords the key advantage of enzyme re-use, and can also enhance their operational and/or storage stability (Cao and Moo-Young, 2011). Among various immobilization techniques available, while the use of covalent binding to solid insoluble carriers has extensively appeared in the literature (Novick et al., 2005), its use within the food sector is relatively under-developed.

The main advantage of the covalent approach is the strength of enzyme binding to a solid phase, theoretically minimizing in-process enzyme leachate from the carrier (Mohy Eldin et al., 2011). Natural polymers such as alginate beads have received considerable attention due to their potential applications in the food and pharmaceutical industries (Martău et al., 2019). The continued search for adoption of natural materials in food processing has also pointed to the exploitation of genipin (from gardenia fruit *Gardenia jasminoides*) as a potentially safer alternative to the conventional crosslinker, glutaraldehyde, for activation of alginate beads prior to covalent binding to enzymes (Tacias-Pascacio et al., 2019). To the best of our knowledge, enzyme immobilization with genipin-activated alginate beads for juice clarification has received little attention. Thus, this work investigates the covalent coupling of pectinase and xylanase to genipin-activated alginate beads for application in apple juice clarification. The immobilized enzyme preparations were subsequently evaluated in terms of reusability and operational-storage stability.

For process optimization purposes, a one-factor-at-a-time (OFAT) approach is a traditional choice which requires changing only one factor at any given time and keeping all other factors constant. Nevertheless, the need for a non-laborious approach that considers the combined interaction of the factors calls for the use of model-based optimization, employing a statistical approach. Nonetheless, a statistical approach does not serve the purpose of optimization for complex, and nonlinear systems. Therefore, an artificial neural network (ANN) is proposed as a computational modeling technique that offers prominent advantages over statistical modeling techniques in capturing non-predefined relationships and non-linearity behaviour in complex systems (Sargent, 2001). Moreover, multiple studies have demonstrated that computational modeling (e.g. artificial neural network) is more accurate than statistical modeling (e.g. response surface methodology) in enzyme immobilization and juice clarification processes (Talib et al., 2019; Youssefi et al., 2009). Hence, an artificial neural network was employed to achieve the maximum recovery of enzyme fractional activity (%) of pectinase and xylanase, as well as maximum apple juice clarification using the immobilized enzymes. For instance, trained ANN model has been successfully employed to optimize the immobilization process of lipase from *Candida rugosa* on Amberjet® 4200-Cl using a multilayer perceptron (Fatiha et al., 2013). Furthermore, back-propagation algorithm has been employed to optimize the immobilization process of cellulase from *Trichoderma viride* on Eudragit® L-100 (Zhang et al., 2012). Moreover, trained ANN model has been successfully employed to optimize apple juice clarification by ultrafiltration using Bayesian regularization algorithm (GökmenAçar et al., 2009), suggesting that incorporation into the present study could be beneficial, The results of the algorithms were compared by minimized root mean squared error (RMSE) and maximized coefficient of determination (R^2^).

## 2 Material and methods

### 2.1 Enzymes

Pectinase (912 U/ml) and xylanase (455 U/ml) were produced from *Mucor hiemalis* AB1 (GenBank accession number: JQ912672.1) in our laboratory (Hassan et al., 2020). Enzyme activity was determined by the dinitrosalicylic acid (DNS) method of Miller (1959) using pectin (citrus peel) or xylan (beechwood) as standards. Unless stated, all the chemicals used in this work were commercial products of analytical grade (Sigma-Aldrich, Ireland).

### 2.2 Enzyme immobilization

#### 2.2.1 Experimental design and data acquisition for ANN modeling

Optimizations were based on the protocol established by (Khairudin et al., 2015). Experimental design was carried out using STATGRAPHICS Centurion XV software (StatPoint Technologies Inc. Warrenton, VA, USA). A four-factor-five-level central composite rotatable design (CCRD) was used to evaluate the fractional enzyme activity (%) recovered after immobilization. The selected CCRD model consisted of four factors, viz. genipin concentration (%) for alginate bead activation, enzyme loading (U/ml), coupling time (min), and agitation rate (rpm). The factors and their levels were obtained through preliminary tests and based on previous results from the literature (Pal and Khanum, 2011a). Table 1 summarizes the range and levels of the four factors.

**Table 1:** Independence factors and corresponding levels for enzyme immobilization.

Code	Factors	Units	Levels				
			-α	−1	0	+1	+α
A	Genipin	%, w/v	0	3	6	9	12
B	Enzyme load	U/ml	50	150	250	350	450
C	Coupling time	min	30	60	90	120	150
D	Agitation rate	rpm	50	100	150	200	250

The central composite design requires 30 experiments consisted of 16 factorial points, 8 axial points, and 6 central points. The design is rotatable (CCRD) since the axial parameter value is α = F^1/4^ = 2, where F is the number of factorial points (Asghar et al., 2014). Once the experiments were performed, the experimental dataset (30 experiments) were randomly divided into two sets - training set and testing set - whereas experimental values at predicted optimum conditions were used as the validating set.

#### 2.2.2 Covalent immobilization of pectinase and xylanase

Initially, alginate beads were prepared by manually dropping sodium alginate solution (3%, w/v) into the hardening solution (calcium chloride, 0.2 M) using a peristaltic pump (Bhushan et al., 2015). The beads were collected using a filter funnel through a Whatman® (No. 1) paper after 3 h of gentle stirring on a magnetic stirrer, and maintained in the gelling solution (CaCl_2_, 0.02 M) overnight at 4 °C to harden. Afterwards, the beads were washed with deionized water and further activated by mixing with genipin solutions of varying concentrations, ranging from 3 to 12% (w/v) in citrate buffer (0.05 M, pH 5.0), and gently stirred to ensure a homogeneous coating of cross-linker. Finally, the beads were removed by filtration and washed with distilled water to remove the unbound genipin. The resulting activated beads were used as carrier in enzyme immobilization experiments.

The immobilization of pectinase and xylanase was performed by orbital mixing (50–250 rpm) of an equal volume (1:1 ratio) of enzyme solution (50–450 IU/ml) with activated beads for different durations (30–150 min). The beads were removed by filtration and washed with distilled water until no enzyme activity could be detected in the washings. The fractional enzyme activity (FEA, %) recovered after immobilization was calculated using the following equation (Zhou et al., 2013):

Fractional enzyme activity (%) = (A ÷ A_init_) ∗ 100

where A is the activity of immobilized enzyme on beads and A_init_ is the initial (free) enzyme activity.

#### 2.2.3 Evaluation of the immobilized enzymes

The optimum reaction pH of the immobilized enzymes was measured in the range between 2.0 and 11.0 using glycine-HCl buffer (0.1 M, pH 2.0), citrate buffer (0.1 M, pH 3.0–6.0), phosphate buffer (0.1 M, pH 7.0–8.0) and glycine-NaOH buffer (0.1 M, pH 9.0–11.0); and the optimum temperature was measured in the range between 20 °C and 80 °C. To evaluate the storage stability, immobilized enzymes were held for 30 days at 4 °C. For a reusability assessment, immobilized enzymes were recovered by magnetic separation after each cycle of use and washed with deionized water, and then a new cycle was run under the same conditions for a total of 6 cycles. The enzyme activity in the first cycle was assigned a value of 100%, and relative activity was calculated for the successive cycles. All experiments were performed in triplicate.

### 2.3 Enzymatic treatment of apple juice

#### 2.3.1 Experimental design and data acquisition for ANN modeling

A four-factor-five-level central composite rotatable design (CCRD) that required 30 experiments was used to evaluate the juice clarification (%). The selected CCRD model consisted of four factors, viz. pectinase loading (U/ml of apple juice), xylanase load (U/ml of apple juice), holding time (min), and temperature (°C). The factors and their levels were obtained through preliminary tests and based on previous results from the literature (Ravindran et al., 2019). Table 2 summarizes the range and levels of the four factors.

**Table 2:** Independence factors and corresponding levels for clarification of apple juice.

Code	Variables	Units	levels				
			-α	−1	0	+1	+α
A	Pectinase load	U/ml of apple juice	10	20	30	40	50
B	Xylanase load	U/ml of apple juice	10	20	30	40	50
C	Holding time	min	40	60	80	100	120
D	Temperature	°C	40	45	50	55	60

The CCRD contained 16 factorial points, 8 axial points, and 6 central points, with α value fixed at 2.0 for a total of 30 experiments. Once the experiments were performed, the experimental dataset (30 experiments) were randomly divided into two sets - training set and testing set - while experimental values at predicted optimum conditions were used as the validating set.

#### 2.3.2 Clarification of apple juice using immobilized enzymes

Fresh apple fruits (*Malus domestica*) of Royal Gala variety (without any visual defects) at commercial maturity were purchased from a local market (Dublin, Ireland). The apples were washed with tap water, chopped into small pieces, and later macerated in a domestic blender, without addition of water, until a homogenous juice was obtained. The concentrated juice was then pasteurized for 1 h at 60 °C (Naga Padma et al., 2017). The filtered juice (pH 5.0) was used for the clarification studies.

**Fig. 1:**
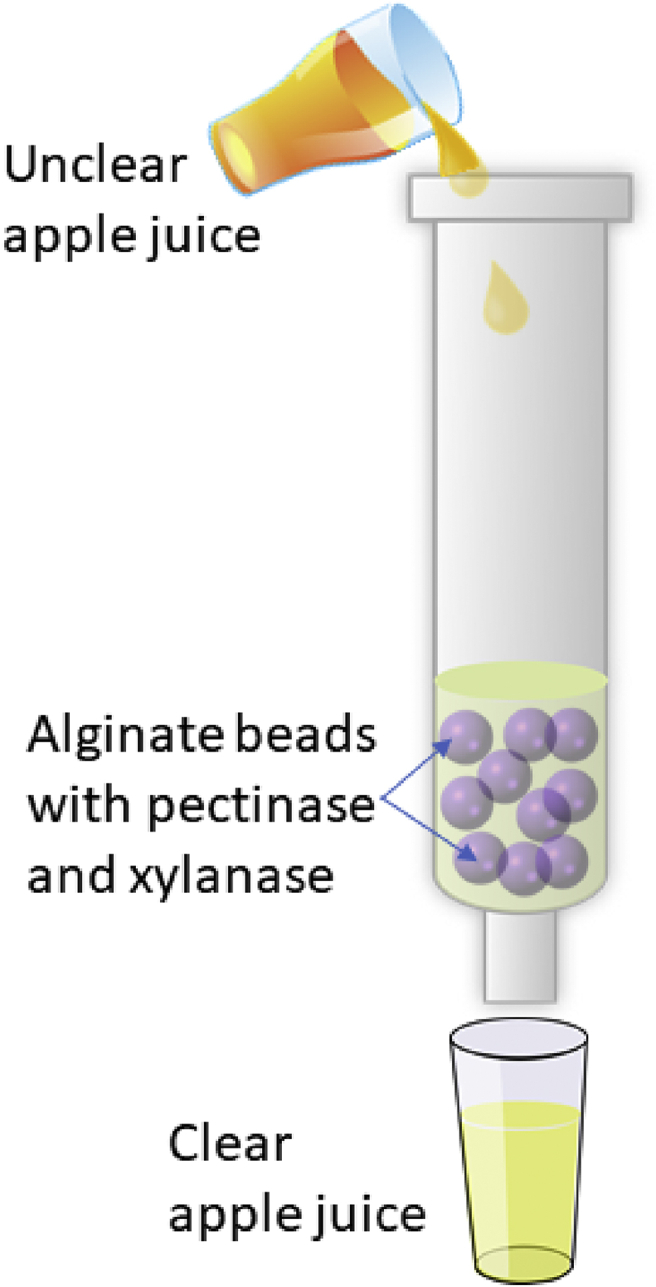
Schematic diagram of the experimental set-up for clarification of apple juice.

Fig. 1 illustrates the laboratory scale set up of a packed-bed reactor using a glass column for enzymatic clarification of apple juice using the immobilized enzymes (pectinase and xylanase) on alginate beads. The enzymatic clarification experiments were performed by subjecting 25 ml of apple juice to different concentrations of pectinase and xylanase (10–50 U/ml of juice) for varying duration of holding times (40–120 min) within the range of temperatures between 40 °C and 60 °C. Finally, the enzyme beads were removed, and treated apple juice centrifuged (10,000 rpm, 15 min), followed by filtration using Whatman no 1 filter paper, and this juice filtrate was used for further analysis. The clarity of juice was expressed as percentage transmission (%T) that was determined using a UV-1800 UV-VIS spectrophotometer (Shimadzu Scientific Instruments, Columbia, USA) at a wavelength of 660 nm, and using distilled water as a reference (Dey and Banerjee, 2014).

### 2.4 Artificial neural network (ANN) modeling and analysis

The commercial artificial intelligence software, NeuralPower® (CPC-X Software, version 2.5, Carnegie, PA, USA) was employed for ANN modeling and analysis. The networks were trained in a supervised learning environment by different learning algorithms (incremental back propagation, IBP; batch back propagation, BBP; quickprob, QP; and Levenberg-Marquardt algorithm, LM). Multilayer normal feed-forward was used to predict the response and the hyperbolic tangent function (a.k.a. tanh) used as transfer function in the hidden and output layers. To determine the optimal network topology, only one hidden layer with varying number of neurons was used to develop different networks. The comparison between the models were assessed using root mean square error (RMSE) and correlation coefficient (R^2^). Models were further assessed using a testing dataset to predict the unseen data (data not used for ANN training). For process optimization, three different optimization algorithms were employed, namely rotation inherit optimization (RIO), particle swarm optimization (PSO), and genetic algorithm (GA). After determination of optimum conditions, experimental validation was carried out to calculate the percentage error between the experimentally measured values and the ANN predicted value using the formula (Zhang et al., 2020) as follows:

Error (%) = [(P′-P)/P] ∗100

where, P′ is the ANN-predicted recovery of enzyme fractional activity, and P is the observed recovery of enzyme fractional activity measured in the experiment.

## 3 Results and discussion

### 3.1 Enzyme immobilization

#### 3.1.1 The ANN model training

A neural network with optimal number of neurons is required to avoid over- or undertraining of the training dataset. If neurons are lower than the optimum range, undertraining would result in a poor fit to the training dataset. On the other hand, increasing the number of hidden neurons above the optimum range may lead to overfitting, as the network may end up memorizing the training data. Although this would result in very good fit to the training dataset, the model would have poor generalization ability to handle testing and unseen datasets.

**Fig. 2:**
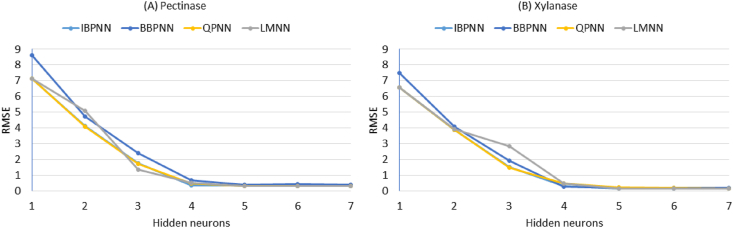
The performance of different learning algorithms (Incremental backpropagation algorithm, IBP; Batch backpropagation algorithm, BBP; Quick propagation algorithm, QP; and Levenberg-Marquardt algorithm, LM) for training data versus of the number of neurons in the hidden layer for predicting the recovery of enzyme fractional activity of pectinase (A) and xylanase (B) onto alginate beads.

The larger subset (n = 25) comprising more than 80% of the available experimental data was used for the ANN training and model building. To determine the optimal topology for the networks, the number of neurons in the hidden layer was varied from 1 to 7. Subsequently, the decision on the optimum topology was based on the minimum RMSE (and the closer R^2^ to 1) of testing set values. Fig. 2 illustrates the performance of the network for testing data versus of the number of neurons in the hidden layer using different learning algorithms.

**Fig. 3:**
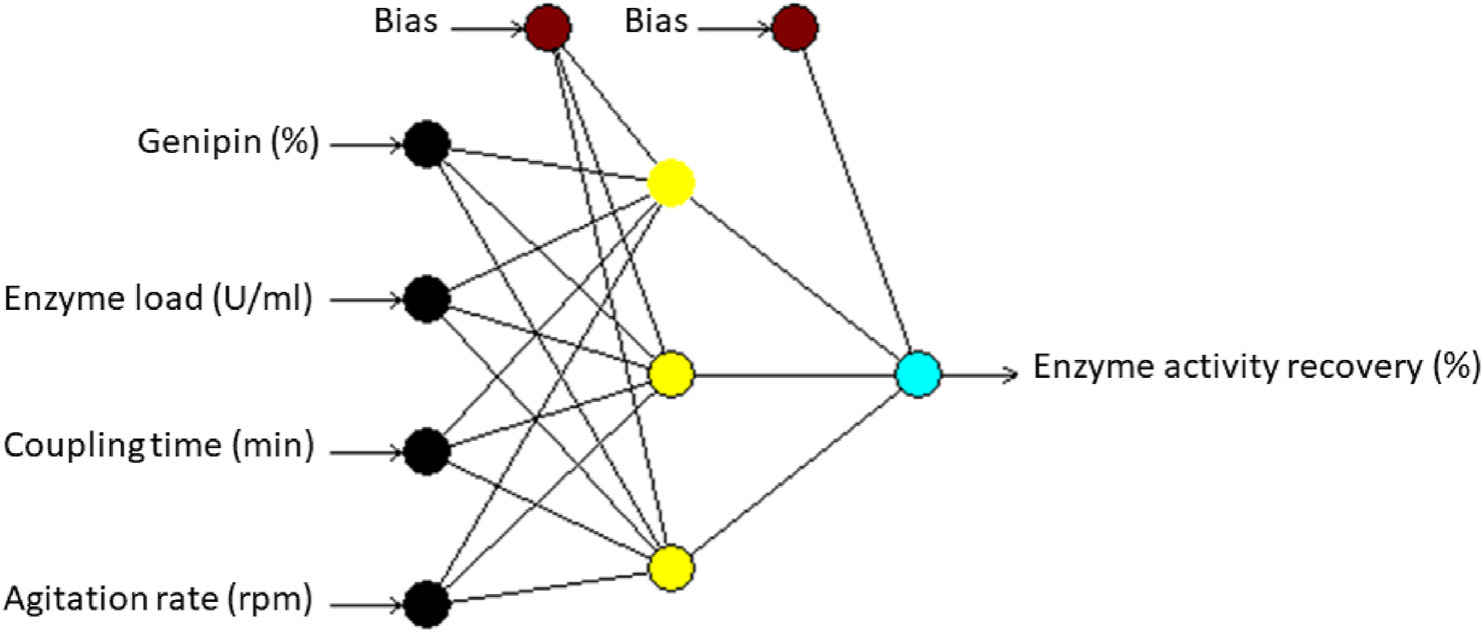
The illustration of multilayer normal feed-forward neural network. The neural network having three inputs of variables (genipin, enzyme load, coupling time, and agitation rate), one hidden layer with three neurons (nodes) and one output of response (enzyme activity recovery).

According to the RMSE, the network with 3 hidden neurons produced the optimum performance when any of the four algorithms (IBP, BBP, QP and LM) was employed. Therefore, the optimum topology of the networks (Fig. 3) was 4-3-1 (four neurons in the input layer, three neurons in the hidden layer and one neuron in the output layer).

#### 3.1.2 Selection neural network model

**Fig. 4:**
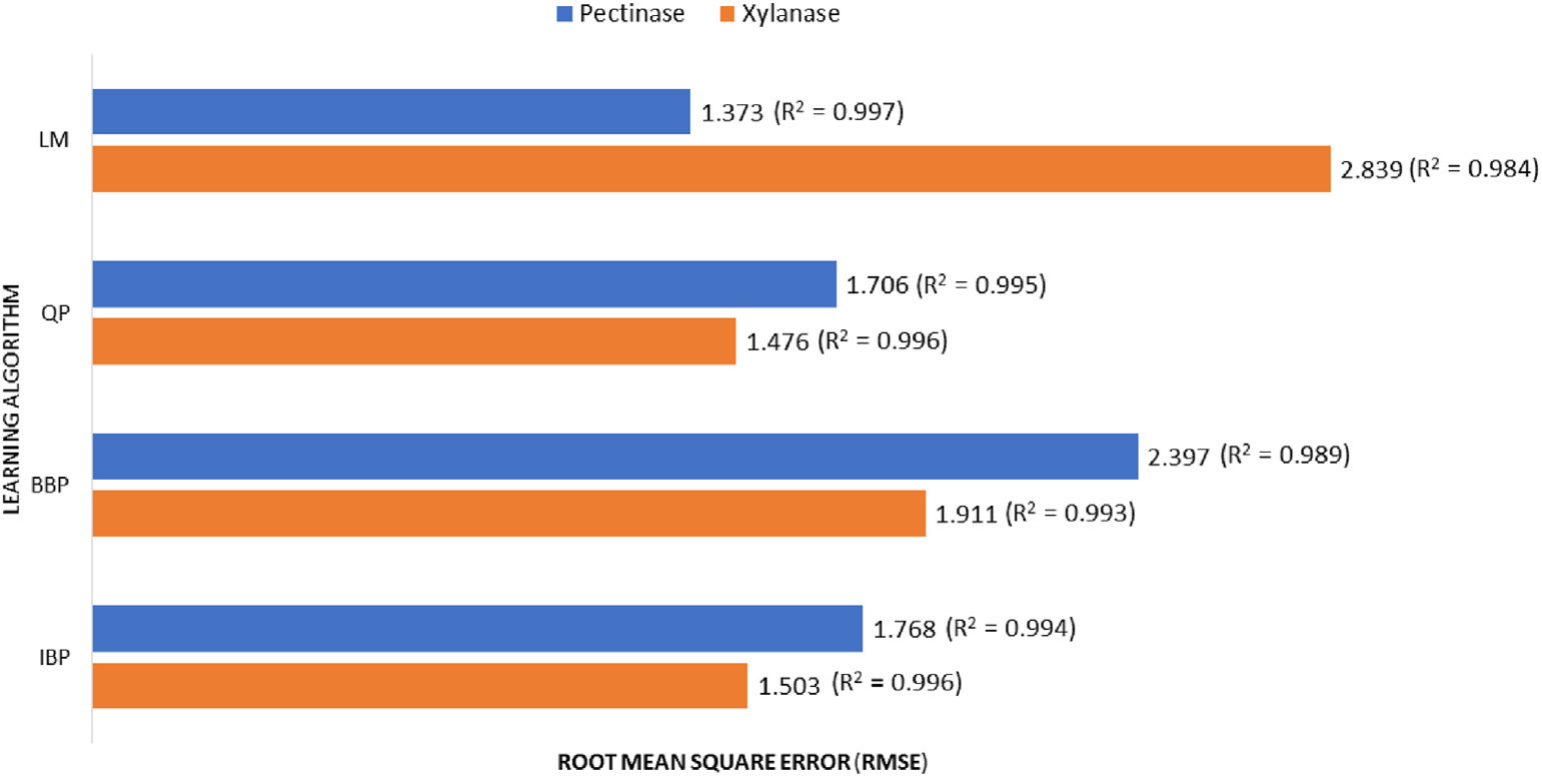
Comparison of different learning algorithms (Incremental backpropagation algorithm, IBP; Batch backpropagation algorithm, BBP; Quick propagation algorithm, QP; and Levenberg-Marquardt algorithm, LM) with 6 neurons in the hidden layer for predicting the recovery of enzyme fractional activity of pectinase and xylanase onto alginate beads.

The model architecture of 4-3-1 was selected as the best topology for the four learning algorithms. Moreover, as shown in Fig. 4, LM and QP were at maximum R^2^, while its RMSE were at the lowest value in comparison with the other algorithms for predicting the recovered fractional enzyme activity (%) of pectinase and xylanase, respectively. Backpropagation is an extensively used family of supervised training algorithms based on the error-correction learning rule and can be implemented in either incremental or batch mode (Kahraman, 2012). Backpropagation algorithm has been improved for a faster training process (‘quick propagation- QP- algorithm’ (Awolusi et al., 2018);), and enhanced performance (‘Levenberg Marquardt – LM - algorithm’ (Reddy et al., 2018);). It was reported that QP gave the best performance in modeling the enzymatic synthesis of betulinic acid ester when compared with IBP, BBP, and LM (Moghaddam et al., 2010). On the other hand, Adnani et al. (2011) employed the LM algorithm for lipase-catalyzed synthesis of sugar alcohol ester. It is worth noting that there is no ideal algorithm *per se* that will give the best results in the training of any dataset, and the result of training is highly dependent on the architecture of the network, the training algorithm, the size of training dataset and data noise levels (Jacobsson, 1998).

Table 3 displays the ANN predicted values for the training datasets using LM-4-3-1 for pectinase immobilization and QP-4-3-1 for xylanase immobilization. The results revealed the close correlation between the experimental and the predicted values. The R^2^ and RMSE metrics were used to evaluate the developed models. The R^2^ value was 0.99 for both models, where RMSE values were 1.37 and 1.48 for pectinase and xylanase immobilization models, respectively. The obtained R^2^ of the two models is very close to 1, indicating a good adjustment between the observed and predicted values. Moreover, the obtained low RMSE values of the two models did not show significant disparity, indicating relatively similar performance.

**Table 3:** Experimental design showing the observed and predicted recovery values of fractional enzyme activity (%) as output for training dataset.

Run	Independent Variables				Response			
	A	B	C	D	Fractional enzyme activity (%)			
					Pectinase		Xylanase	
					Observed	Predicted	Observed	Predicted
Training Data								
1	3	150	60	100	18.66	16.96	16.86	16.77
2	3	150	120	200	62.25	63.77	61.51	61.52
3	6	250	90	150	31.04	31.93	30.81	31.95
4	6	50	90	150	60.44	60.37	61.12	61.30
5	12	250	90	150	42.95	42.95	41.16	42.49
6	9	350	120	200	25.49	25.85	26.24	26.08
7	9	350	60	100	36.24	36.04	34.66	35.14
8	3	350	120	200	31.89	31.55	29.22	28.00
9	3	150	60	200	26.98	25.53	26.99	26.28
10	9	350	120	100	36.49	36.21	35.91	34.26
11	6	250	30	150	30.64	30.67	28.48	25.97
12	3	350	120	100	20.56	20.56	21.94	21.50
13	9	150	60	100	42.5	44.18	44.35	46.67
14	6	450	90	150	18.83	18.83	16.3	16.85
15	0	250	90	150	10.41	12.34	12.00	13.50
16	6	250	90	150	32.01	31.93	31.27	31.95
17	3	350	60	100	15.92	13.37	17.28	20.27
18	6	250	90	50	41.81	41.84	40.68	37.55
19	6	250	90	150	31.92	31.93	31.23	31.95
20	9	150	120	200	83.26	80.23	80.53	79.01
21	6	250	90	150	33.19	31.93	31.83	31.95
22	3	350	60	200	15.85	18.87	16.04	14.55
23	9	150	60	200	51.61	52.33	48.53	46.75
24	6	250	90	150	31.74	31.93	31.14	31.95
25	6	250	90	250	43.75	42.32	42.86	43.83
R^2^						0.99		0.99
RMSE						1.37		1.48

A subset (n = 5) comprising just above 15% of the available experimental data was used for ANN testing to predict the unseen data (data not used for ANN training). Hence, the trained ANNs was tested against the corresponding testing datasets to assess the predictive power of the developed ANN models. Table 4 displays the ANN predicted values for the testing datasets using LM-4-3-1 for pectinase immobilization and QP-4-3-1 for xylanase immobilization. The R^2^ value was 0.99 for both models, where RMSE values were 1.73 and 1.86 for pectinase and xylanase immobilization models, respectively. The obtained R^2^ indicated that the regression predictions perfectly fit the data. In addition, the obtained RMSE values showed a small difference between training and testing datasets (0.36 for pectinase immobilization model, and 0.38 for xylanase immobilization model), indicating good generalization capability and accuracy of the trained ANN models.

**Table 4:** Experimental design showing the observed and predicted recovery values of fractional enzyme activity (%) as output for testing dataset.

Run	Independent Variables				Response			
	A	B	C	D	Fractional enzyme activity (%)			
					Pectinase		Xylanase	
					Observed	Predicted	Observed	Predicted
Testing Data								
1	3	150	120	100	46.07	45.78	44.02	42.33
2	0	250	250	150	64.49	62.56	63.45	61.40
3	6	250	90	150	32.41	31.93	31.46	32.01
4	9	150	120	100	75.91	73.89	74.56	72.35
5	9	350	60	200	15.92	13.62	17.45	18.68
R^2^						0.99		0.99
RMSE						1.73		1.86

#### 3.1.3 Optimization of enzyme immobilization process using trained ANNs

**Fig. 5:**
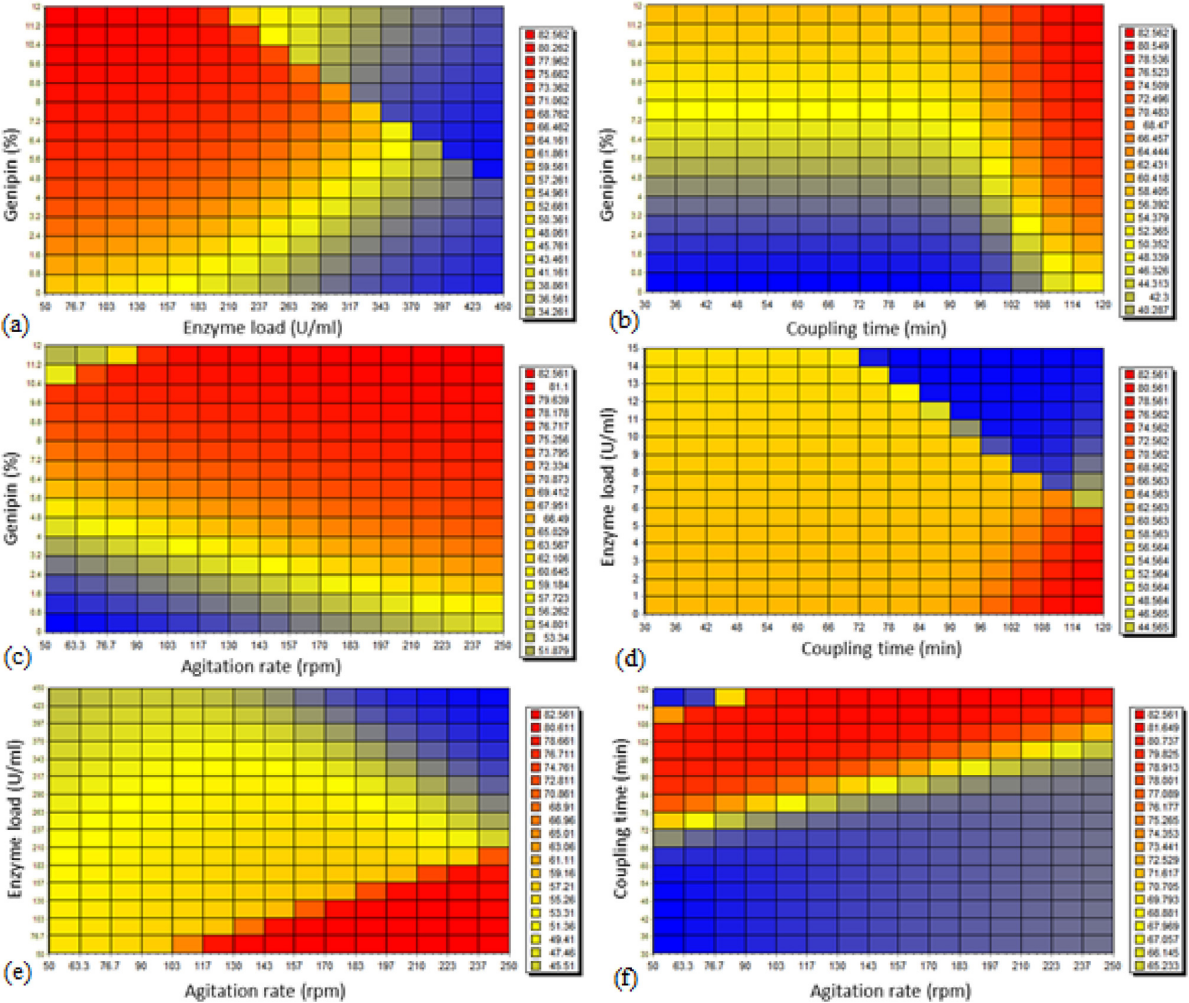
Grid color charts representing the effect of independent variables on recovery of pectinase enzyme fractional activity (%): (a) Effect of genipin concentration and pectinase load on enzyme fractional activity recovered when coupling time and agitation rate are fixed at 120 min and 213 rpm, respectively; (b) Effect of genipin concentration and coupling time on enzyme fractional activity recovered when pectinase load and agitation rate are fixed at 50 U/ml and 213 rpm, respectively; (c) Effect of genipin concentration and agitation rate on enzyme fractional activity recovered when pectinase load and coupling time are fixed at 50 U/ml and 120 min, respectively; (d) Effect of pectinase load and coupling time on enzyme fractional activity recovered when genipin concentration and agitation rate are fixed at 12% (w/v) and 213 rpm, respectively; (e) Effect of pectinase load and agitation rate on enzyme fractional activity recovered when genipin concentration and coupling time are fixed at 12% (w/v) and 120 min, respectively; and (f) Effect of coupling time and agitation rate on enzyme fractional activity recovered when pectinase load and genipin concentration are fixed at 50 U/ml and 12% (w/v), respectively.

**Fig. 6:**
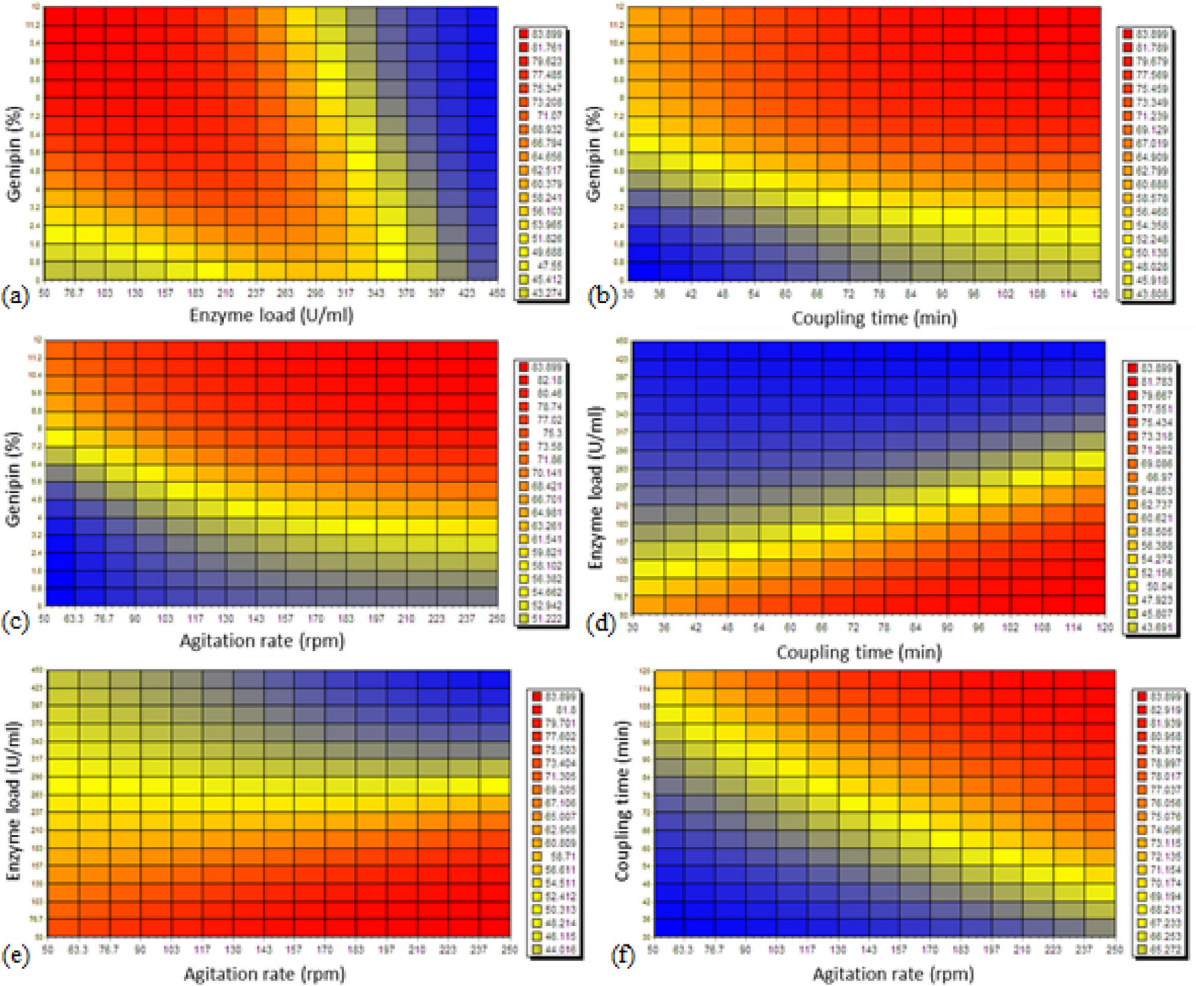
Grid color charts representing the effect of independent variables on recovery of xylanase enzyme fractional activity (%): (a) Effect of genipin concentration and xylanase load on enzyme fractional activity recovered when coupling time and agitation rate are fixed at 120 min and 250 rpm, respectively; (b) Effect of genipin concentration and coupling time on enzyme fractional activity recovered when xylanase load and agitation rate are fixed at 50 U/ml and 250 rpm, respectively; (c) Effect of genipin concentration and agitation rate on enzyme fractional activity recovered when xylanase load and coupling time are fixed at 50 U/ml and 120 min, respectively; (d) Effect of xylanase load and coupling time on enzyme fractional activity recovered when genipin concentration and agitation rate are fixed at 12% (w/v) and 250 rpm, respectively; (e) Effect of xylanase load and agitation rate on enzyme fractional activity recovered when genipin concentration and coupling time are fixed at 12% (w/v) and 120 min, respectively; and (f) Effect of coupling time and agitation rate on enzyme fractional activity recovered when xylanase load and genipin concentration are fixed at 50 U/ml and 12% (w/v), respectively.

The optimum conditions for enzyme (pectinase and xylanase) immobilization were determined by comparing three different algorithms, which were rotation inherit optimization (RIO), particle swarm optimization (PSO), and genetic algorithm (GA). However, there was no significant difference in values of fractional enzyme activity (%) predicted by the three different algorithms. The predicted conditions for optimum pectinase fractional activity (82.56%) were 50 U/ml xylanase with 12% of genipin crosslinker, with a coupling time of 120 min and agitation rate of 213 rpm. Similarly, the predicted conditions for optimum xylanase fractional activity (83.89%) were also 50 U/ml xylanase with 12% of genipin crosslinker and coupling time of 120 min, but with an agitation rate of 250 rpm. The grid color charts of pectinase and xylanase fractional activity (%) are shown in Fig. 5, Fig. 6, respectively.

Pal and Khanum (2011a) reported a slightly higher RSM-predicted recovery of xylanase fractional activity (89.5%) on alginate beads compared to our ANN-predicted values (84%) using 8.31% gluterdehyde crosslinker, 250 U/ml of xylanase from *Aspergillus niger*, coupling time of 120 min and an agitation rate of 200 rpm. On the other hand, Abdel Wahab et al. (Abdel Wahab et al., 2018) reported a slightly lower RSM-predicted recovery of fractional activity for pectinase (80.43%) comparing to our ANN-predicted values (83%) using 5% polyethyleneimine (PEI), 1.5% gluteraldehyde, 15 U/ml of pectinase from *Aspergillus awamori*, and a coupling time of 6 h.

From Fig. 5, Fig. 6a, it is observed that with increase in genipin concentration, immobilization efficiency will also theoretically increase, as more attachment points become available for enzyme immobilization on the alginate beads. However, increasing the enzyme load (pectinase or xylanase) will not always lead to an increase in immobilization efficiency, presumeably due to insufficient attachment points of genipin for enzyme. Similar results were reported by Sukri and Munaim (2017) where the highest recovery of xylanase fractional activity was achieved when the alginate beads were activated by a higher concentration of glutaraldehyde and a lower enzyme loading. As one might expect, a longer coupling time and higher genipin concentration resulted in higher immobilization efficiency at constant enzyme loading, as shown in Fig. 5, Fig. 6b. On the other hand, longer coupling time and higher enzyme loading did not result in higher immobilization efficiency at constant genipin concentration, as shown in Fig. 5, Fig. 6d.

The effect of agitation rate on the immobilization efficiency (Fig. 5, Fig. 6c) was less significant compared to the effect of other variables, most probably as mixing serves the single purpose of generating a homogeneous suspension of bead- and enzyme solution. Such a homogeneous suspension improves contact of the free enzyme with the beads, which results in higher immobilization efficiency. Hence, as the agitation rate increases above the optimum rate, immobilization efficiency shows no significant improvement. Sukri et al. (Sabrina et al., 2020) reported an equivalent result, reporting that an agitation rate of 200 rpm resulted in an optimum recovery of fractional activity (83.93%) of xylanase (200 U) on alginate beads activated by glutaraldehyde (12%, w/w), but no improvements could be achieved in using a higher rate.

The model validation was carried out by running at the predicted conditions (Table 5). As a result of three successive runs, only slight variation (1.5–1.6%) in the value of recovered enzyme fractional activity was observed, suggesting that the optimal recovery conditions for enzyme fractional activity of both enzymes generated by the ANN algorithms were reliable and valid.

**Table 5:** Experimental validation of the optimization values predicted by ANN for recovery of fractional enzyme activity (%).

Replicates	Pectinase		Xylanase	
	Predicted	Observed	Predicted	Observed
1	82.56	80.57	83.89	82.56
2		81.96		81.92
3		81.45		83.14
	Mean	81.33	Mean	82.54
	Error (%)	1.52	Error (%)	1.64

#### 3.1.4 Evaluation of the immobilized enzymes

**Fig. 7:**
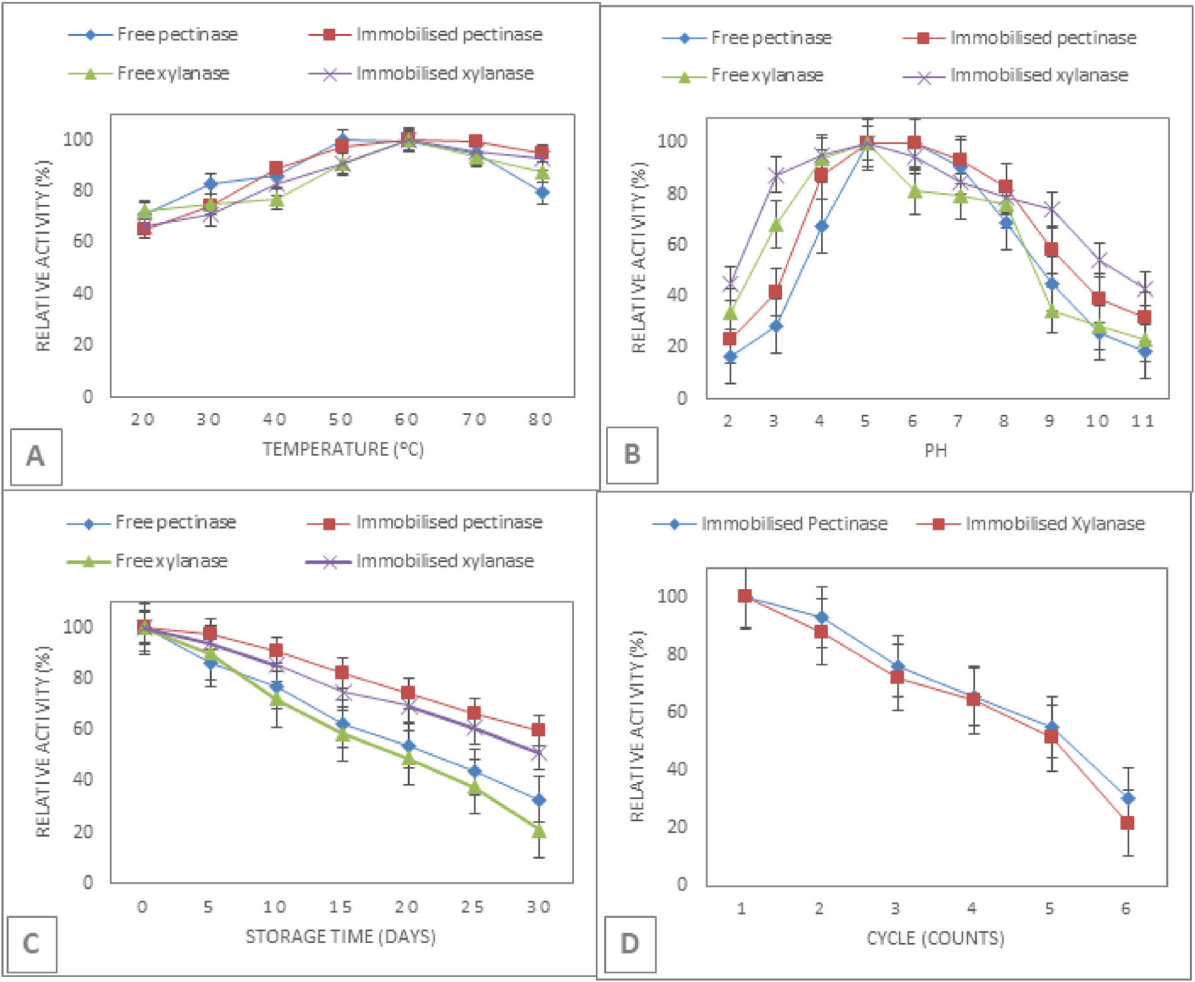
Panels (A–C) shows the effect of temperature (A), pH (B), storage time (C), and recycle count (D) on relative activity of immobilized pectinase and xylanase in comparison with free enzyme.

Immobilized enzymes were evaluated by studying their key required operational parameters (temperature, pH, storage and recycle stability) in comparison with free forms (Fig. 7). As shown in Fig. 7a, the immobilization did not change the optimal temperature of xylanase (60 °C) at pH 5. However, the optimum temperatures of the free and immobilized pectinase were 50 °C and 60 °C, respectively. Such a forward shift in the temperature optimum of immobilized pectinase by 10 °C could be the result of improved enzyme rigidity after covalent binding on alginate beads (Ortega et al., 2009). To study the pH-dependent activities of the free and immobilized enzymes, the temperature of assay mixtures was maintained at 60 °C while pH values were varied from 2.0 to 11.0 (Fig. 7b). Although the immobilized enzymes retained the optimal pH (5.0) of their free pectinase and xylanase counterparts, the pH scope of the immobilized pectinase was expanded, and it retained more than 80% activity over a wider pH range of 4.0–8.0 than that of the free form (pH range of 5.0–7.0). Similarly, the immobilized xylanase exhibited improved pH stability, and retained greater than 80% activity over a wider pH range of 3.0–7.0 than that of the free form (pH range of 4.0–6.0). These results may be attributed to the free protein undesired aggregation that is prevented by the covalent bonding of the enzyme onto alginate beads during the immobilization process (Mostafa et al., 2019).

The data in Fig. 7c show the storage stability of enzymes immobilized onto alginate beads over 30 days at 4 °C. Immobilized pectinase and xylanase retained 60% and 51%, respectively, of their initial activity after 30 days of storage. In contrast, the free pectinase and xylanase lost more than 46% and 51%, respectively, of their initial activity after 20 days of storage. The multiple re-use capability of immobilized enzymes can be achieved by recovering the beads from the reaction mixture, thereby reducing costs. As shown in Fig. 7d, the residual activity of the immobilized enzymes was 55% (pectinase) and 51% (xylanase), after five consecutive cycles. The activity loss of the immobilized enzyme may be due to a combination of inactivation and enzyme leakage from the support (Mohamad et al., 2015).

### 3.2 Enzymatic treatment of apple juice

#### 3.2.1 The ANN model training

**Fig. 8:**
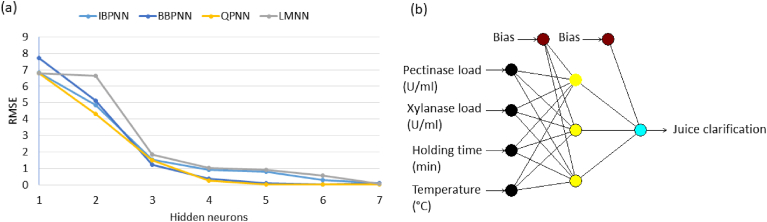
The left panel (a), shows the performance of different learning algorithms (Incremental backpropagation algorithm, IBP; Batch backpropagation algorithm, BBP; Quick propagation algorithm, QP; and Levenberg-Marquardt algorithm, LM) for training data versus of the number of neurons in the hidden layer for predicting the apple juice clarification (%) using pectinase and xylanase immobilized onto alginate beads. The right panel (b), shows schematic diagram of the optimal multi-layer, normal feed-forward neural network architecture for apple juice clarification.

The larger subset (n = 25) comprising more than 80% of the available experimental data was used for the ANN training and model building. To determine the optimal topology for the networks, the number of neurons in the hidden layer was varied from 1 to 7. A decision on the optimum topology was subsequently based on the minimum RMSE (and the closer R^2^ to 1) of the testing set values. Fig. 8a illustrates the performance of the network for testing data versus the number of neurons in the hidden layer using different learning algorithms. According to the RMSE, the network with 3 hidden neurons produced the optimum performance when any of the four algorithms (IBP, BBP, QP and LM) was employed. Therefore, the optimum topology of the networks (Fig. 8b) was 4-3-1 (four neurons in the input layer, three neurons in the hidden layer and one neuron in the output layer).

#### 3.2.2 Selection neural network model

**Fig. 9:**
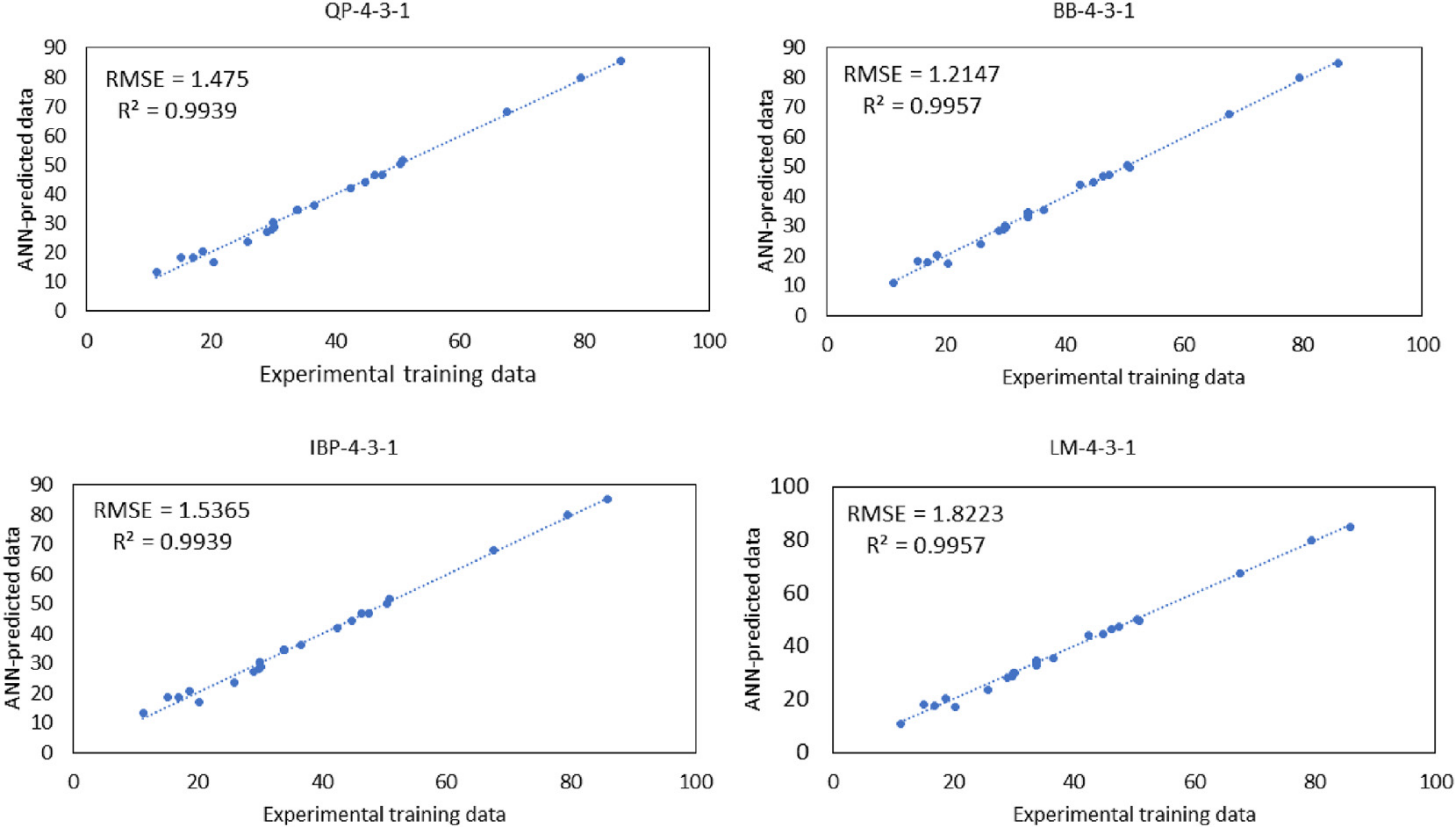
The scatter plots of the predicted juice clarification versus the observed juice clarification for training dataset that shows the performed R^2^ and RMSE of different learning algorithms at optimal neural network architecture (4-3-1).

The model architecture of 4-3-1 was selected as the best topology for the four learning algorithms. Fig. 9 presents the predictions using different learning algorithms with optimum architecture (4-3-1) versus the observed values of the juice clarification (%) which were obtained in the laboratory.

The comparison of the RMSE proved that the BB with 4 nodes in input, 3 nodes in hidden, and 1 node in the output layer (BB-4-3-1) presented the minimum RMSE, while its R^2^ was at the maximum value. As illustrated, the RMSE was 1.21, and the R^2^ was 0.998 which indicated the great predictive accuracy of the model. Therefore, BB-4-3-1 was selected as the final optimum provisional model of the juice clarification for an evaluation test. Table 6 displays the ANN predicted values for the testing datasets using BB-4-3-1 for juice clarification (%). It is worth noting that the batch algorithms are effective in training small datasets with small network topologies (Plagianakos et al., 2001).

**Table 6:** Experimental design showing the observed and predicted values of juice clarification (%) as output for training dataset.

Run	Independent Variables				Response	
	A	B	C	D	Juice clarification (%)	
					Observed	Predicted
Training Data						
1	40	20	60	55	50.46	50.32
2	30	30	80	50	33.79	32.84
3	20	40	100	55	29.96	30.21
4	50	30	80	50	44.79	44.68
5	20	20	60	55	30.19	29.86
6	20	40	60	55	20.27	17.34
7	40	40	60	45	36.48	35.51
8	20	20	100	45	47.46	47.20
9	30	50	80	50	15.14	18.24
10	20	20	100	55	67.49	67.73
11	40	20	60	45	50.84	49.79
12	20	40	100	45	25.75	23.80
13	30	30	80	50	33.79	34.36
14	40	40	100	55	29.69	28.82
15	30	30	80	50	33.79	34.63
16	40	20	100	55	85.80	84.82
17	30	30	40	50	28.97	28.34
18	30	30	80	50	33.79	34.63
19	40	20	100	45	79.37	79.99
20	20	20	60	45	16.96	17.73
21	30	30	80	50	33.79	34.17
22	40	40	60	55	18.59	20.19
23	10	30	80	50	11.19	10.84
24	30	30	80	40	42.48	44.02
25	30	30	80	60	46.31	46.64
R^2^						0.99
RMSE						1.22

A subset (n = 5) comprising just above 15% of the available experimental data was used for ANN testing to predict the unseen data (data not used for ANN training). Hence, the trained ANNs was tested against the corresponding testing datasets to assess the predictive power of the developed ANN models (Table 7). The R^2^ value was 0.99, where RMSE value was 1.84 for the trained ANN. Such an R^2^ value indicates that the regression predictions perfectly fit the data. In addition, the obtained RMSE values showed a small difference between training and testing datasets (0.62), indicating good generalization capability and accuracy of the trained ANN model (BB-4-3-1).

**Table 7:** Experimental design showing the observed and predicted values of juice clarification (%) as output for testing dataset.

Run	Independent Variables				Response	
	A	B	C	D	Juice clarification (%)	
					Observed	Predicted
Testing Data						
1	30	10	80	50	67.02	64.36
2	40	40	100	45	39.09	39.88
3	20	40	60	45	21.18	22.09
4	30	30	120	50	68.87	71.61
5	30	30	80	50	33.79	32.90
R^2^						0.99
RMSE						1.84

#### 3.2.3 Optimization of juice clarification process using trained ANNs

The optimum points for juice clarification were determined by comparing three different algorithms, namely rotation inherit optimization (RIO), particle swarm optimization (PSO), and genetic algorithm (GA). However, there was no significant difference in values of enzyme activity recovery (%) predicted by the algorithms. The predicted conditions for juice clarification (85.62%) were 50 U of pectinase/ml of juice and 20 U of xylanase/ml of juice for 100 min at 57 °C. Thus, our findings are in line with previous literature (Rai et al., 2004; Sin et al., 2006) confirming that enzymatic treatment for juice clarification is greatly influenced by enzyme loading, holding time and temperature. After the experimental validation of the model using the optimization conditions, it was found that the observed value (84.33 ± 0.32%) was close to that predicted (85.62%), suggesting the appropriateness of the developed ANN model.

Similarly, Singh and Gupta (2004) achieved apple juice clarification of 85% (%T_650_) using polygalacturonase from *Aspergillus niger* (15 IU/ml) in the presence of 0.01% gelatin at 45 °C and with a 6 h holding time. The most effective clarification (%T_660_, 97%) was reported by Dey and Banerjee (2014) with 1% polygalacturonase from *Aspergillus awamori* Nakazawa (9.87 U/ml) and 0.4% α-amylase from *A. oryzae* (899 U/ml), in the presence of 10 mM CaCl_2_ at 50 °C and with a 2 h holding time. Also, Dey et al. (2014) reported the maximum transmittance of 93% (%T_660_) in clarified apple juice upon enzymatic treatment using polygalacturonase from *Aspergillus awamori* Nakazawa (9.87 U/ml) at 50 °C and a 2 h holding time.

Additionally, researchers have previously reported that the treatment with xylanase positively contributes to the clarity of apple juice. For instance, the treatment of juice with xylanase (15 IU/g of apple pulp) from *Bacillus pumilus* SV-85S lead to a clarity in terms of % transmittance of approximately 42 (%T_660_) at 40 °C, with a 30 min holding time (Nagar et al., 2012). Adigüzel and Tunçer (2016) reported the maximum transmittance of about 18% (%T_660_) in clarified apple juice upon enzymatic treatment using xylanase from *Streptomyces* sp. AOA40 (12.5 U/ml of apple juice) at 60 °C and a 90 min holding time. Also, Phadke and Momin (2015) reported a maximum transmittance of 20% (%T_650_) in clarified juice upon enzymatic treatment using xylanase from *Bacillus megaterium* (20 U/g of apple pulp) at 37 °C and a 4 h holding time.

Madhu et al. (2015) achieved apple juice clarification of approximately 42%, and 49% (%T_650_) using enzyme cocktails (cellulase, pectinase and xylanase) from *P. exigua* and *A. niger*, respectively, at 60 °C and a 50 h holding time. The study of Pal and Khanum (2011b) explored a synergistic effect of xylanase, pectinase and cellulase to improve clarity of pineapple juice, achieving about 81% (%T_650_) clarity.

**Fig. 10:**
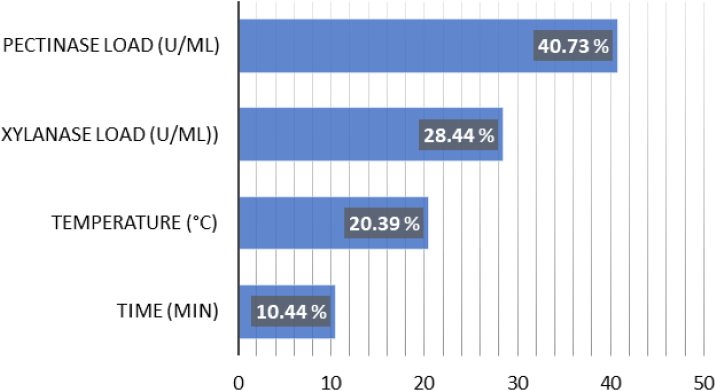
Importance of effective parameters on apple juice clarification.

**Fig. 11:**
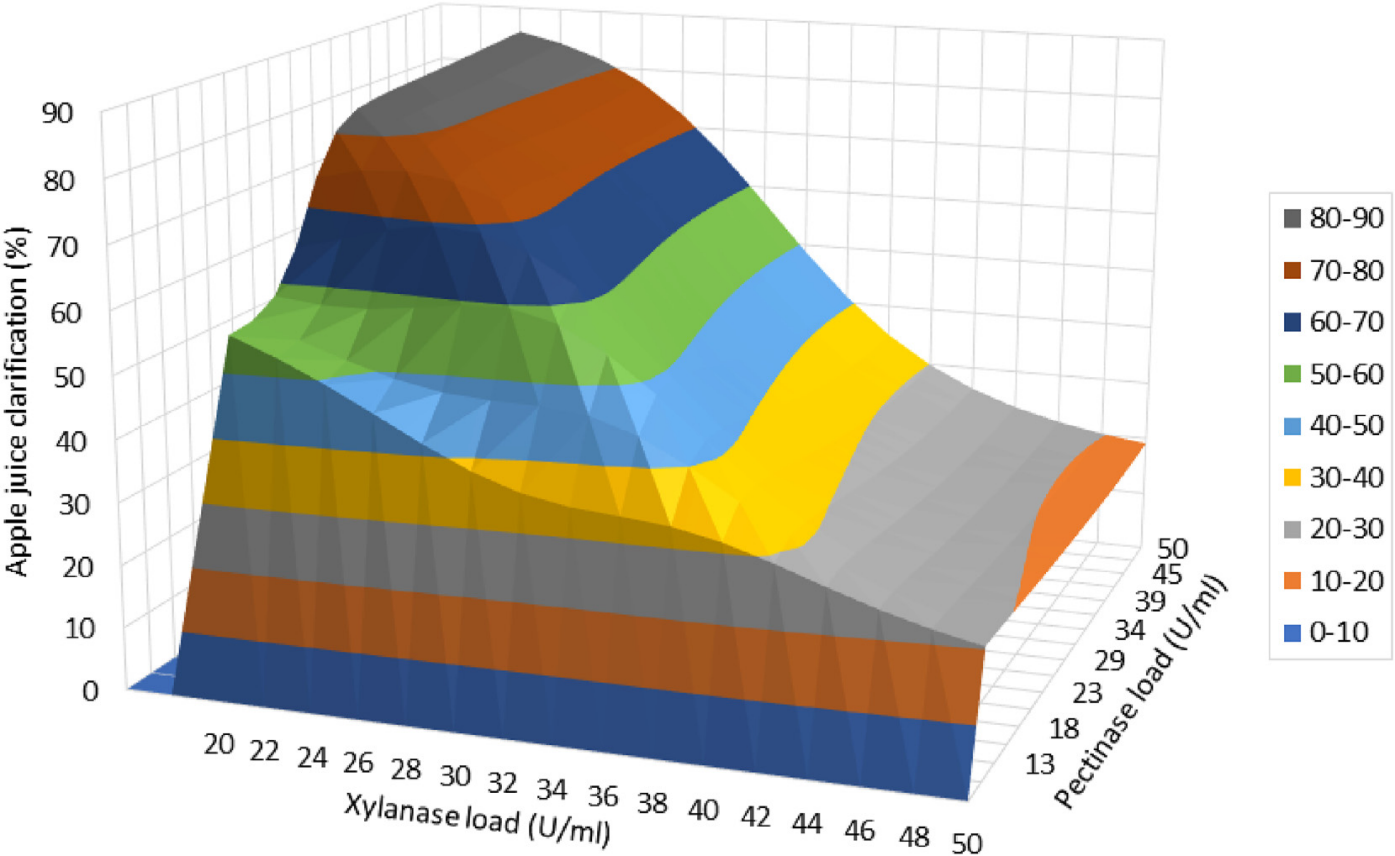
Three-dimensional surface plot of pectinase load and xylanase load effects on apple juice clarification. The other variables (temperature and time) were kept constant at the optimal values.

Fig. 10 demonstrates the importance of effective parameters on apple juice clarification as an output of the model. The importance values of the parameters were pectinase loading > xylanase loading > temperature > holding time in the selected range of the variables. Thus, the effects of the two most important parameters (pectinase and xylanase loadings) on apple juice clarification are presented in Fig. 11, where temperature and time were kept constant at the optimal values (100 min, and 57 °C, respectively). As shown in Fig. 11, apple juice clarity increased with an increase in pectinase rather than xylanase loading at optimum reaction conditions (100 min, and 57 °C). This can be attributed to the fact that apples are particularly rich sources of pectin rather than xylan.

Finally, Table 8 summarizes application data sheets of different commercial enzyme preparations available in the market for apple juice clarification and suggests a role for the use of immobilized enzyme beads developed in our study.

**Table 8:** Application of different commercial enzyme preparations in apple juice clarification.

Company	Product	Description	Dosage	Application			Ref.
				pH	Temp. (°C)	Time (min)	
DSM	RAPIDASE	Pectinases from *A. niger* and *A. aculeatus*	20–40 mL/1000 L of Juice	3.5–5.5	45–55	90–120	Mecti (2015)
Biovet	Pectinase	Pectinesterase, polygalacturonase, pectinlyase from *A. niger*	2–20 g/ton	3.5–6.0	50–55	30–60	Biovet (2020)
Eaton	Panzym Pro Clear	Polygalacturonase from *A. niger* and *A. aculeatus*	20–50 mL/1,000 L of Juice	–	50–55	60–120	Eaton (2015)

## 4 Conclusion

The artificial neural network (ANN) modeling was adopted to simulate and predict the activity recovery of enzymes (pectinase and xylanase) immobilized onto genipin-activated alginate beads, as well as their application in apple juice clarification. The predicted conditions for optimum recovery of pectinase fractional activity (~83%) were 50 U/ml pectinase with 12% of genipin crosslinker and a coupling time of 120 min (agitation rate of 213 rpm). On the other hand, the predicted conditions for optimum recovery of xylanase fractional activity (~84%) were also 50 U/ml xylanase with 12% of genipin crosslinker and coupling time of 120 min, but at an agitation rate of 250 rpm. A maximum recovery of fractional activity was observed to be about 81–83% for both enzymes. Moreover, the predicted conditions for juice clarification (85.62%) were 50 U/ml of pectinase, 20 U/ml of xylanase for 100 min at 57 °C. It was found also that the observed value (84.33 ± 0.32%) was close to that predicted (85.62%). The developed model indicated pectinase loading as the most important factor, having a dramatic influence on apple juice clarification. Enzyme beads prepared at optimum activity recovery conditions were suitable for up to 5 repeated uses, losing only ~45% (pectinase) and ~49% (xylanase) of their initial activity.

## CRediT authorship contribution statement

**Shady S. Hassan**: Conceptualization, Methodology, Resources, Investigation, Data curation, Formal analysis, Software, Visualization, Writing - original draft, Writing - review & editing. **Gwilym A. Williams**: Conceptualization, Validation, Supervision, Project administration, Resources, Funding acquisition, Writing - review & editing. **Amit K. Jaiswal**: Conceptualization, Validation, Supervision, Project administration, Resources, Funding acquisition, Writing - review & editing. All authors have read and agreed to the published version of the manuscript.

## Declaration of competing interest

The authors declare that they have no known competing financial interests or personal relationships that could have appeared to influence the work reported in this paper.
